# Bone-Metabolism-Related Serum microRNAs to Diagnose Osteoporosis in Middle-Aged and Elderly Women

**DOI:** 10.3390/diagnostics12112872

**Published:** 2022-11-19

**Authors:** Sheng-Li Zhao, Zhen-Xing Wen, Xiao-Yi Mo, Xiao-Yan Zhang, Hao-Nan Li, Wing-Hoi Cheung, Dan Fu, Shi-Hong Zhang, Yong Wan, Bai-Ling Chen

**Affiliations:** 1 Department of Spine Surgery, The First Affiliated Hospital of Sun Yat-sen University, Guangzhou 510080, China; 2 Guangdong Provincial Key Laboratory of Orthopaedics and Traumatology, Guangzhou 510080, China; 3 Department of Orthopaedics, Guangzhou First People’s Hospital, Guangzhou 510180, China; 4 Department of Nutrition, School of Public Health, Sun Yat-sen University, Guangzhou 510080, China; 5 Department of Orthopaedics and Traumatology, Prince of Wales Hospital, The Chinese University of Hong Kong, Hong Kong SAR 999077, China; 6 Department of Orthopedics, Kiang Wu Hospital, Macau SAR 999078, China; 7 Department of Laboratry Medicine, The First Affiliated Hospital of Sun Yat-sen University, Guangzhou 510080, China

**Keywords:** postmenopausal osteoporosis, serum, microRNA, diagnosis, epigenetics

## Abstract

**Objective**: Postmenopausal osteoporosis (PMOP), a chronic systemic metabolic disease prevalent in middle-aged and elderly women, heavily relies on bone mineral density (BMD) measurement as the diagnostic indicator. In this study, we investigated serum microRNAs (miRNAs) as a possible screening tool for PMOP. **Methods**: This investigation recruited 83 eligible participants from 795 community-dwelling postmenopausal women between June 2020 and August 2021. The miRNA expression profiles in the serum of PMOP patients were evaluated via miRNA microarray (six PMOP patients and four postmenopausal women without osteoporosis (n-PMOP) as controls). Subsequently, results were verified in independent sample sets (47 PMOP patients and 26 n-PMOP controls) using quantitative real-time PCR. In addition, the target genes and main functions of the differentially expressed miRNAs were explored by bioinformatics analysis. **Results**: Four highly expressed miRNAs in the serum of patients (hsa-miR-144-5p, hsa-miR-506-3p, hsa-miR-8068, and hsa-miR-6851-3p) showed acceptable disease-independent discrimination performance (area under the curve range: 0.747–0.902) in the training set and verification set, outperforming traditional bone turnover markers. Among four key miRNAs, hsa-miR-144-5p is the only one that can simultaneously predict changes in BMD in lumbar spine 1–4, total hip, and femoral neck (*β* = −0.265, *p* = 0.022; *β* = −0.301, *p* = 0.005; and *β* = −0.324, *p* = 0.003, respectively). Bioinformatics analysis suggested that the differentially expressed miRNAs were targeted mainly to *YY1*, *VIM*, and *YWHAE* genes, which are extensively involved in bone metabolism processes. **Conclusions**: Bone-metabolism-related serum miRNAs, such as hsa-miR-144-5p, hsa-miR-506-3p, hsa-miR-8068, and hsa-miR-6851-3p, can be used as novel biomarkers for PMOP diagnosis independent of radiological findings and traditional bone turnover markers. Further study of these miRNAs and their target genes may provide new insights into the epigenetic regulatory mechanisms of the onset and progression of the disease.

## 1. Introduction

Postmenopausal osteoporosis (PMOP), which is caused by estrogen withdrawal, is the most frequent type of primary osteoporosis and threatens nearly half of middle-aged and elderly women worldwide [1,2]. Due to the lack of preliminary symptoms and typical features, delayed diagnosis is common in clinical practice, especially in surgical systems [3]. Fragility fracture is one of the most critical complications of PMOP, leading to high disability and mortality. In China alone, the projected cost of osteoporotic fractures may reach USD 25.4 billion by 2050 [4]. Therefore, early detection is essential for alleviating the harm of PMOP.

Bone mineral density (BMD) assessed by dual-energy X-ray absorptiometry (DXA) is widely accepted as an indispensable index for defining PMOP. However, due to differences in development between developed and developing regions in medical service supply, universal access to BMD measurement seems unlikely in the short term [5]. Meanwhile, as a type of assessment method, positive imaging features always lag behind the continuous abnormality of bone metabolism, which weakens the application value of these classical examination methods in diagnosis of the early phase of bone disease [6,7]. As a direct reflection of the changes in bone homeostasis, the re-review of bone turnover markers (BTMs) in recent years seems to provide new hope for the auxiliary diagnosis of PMOP [8,9]. However, recent studies, including our own, have confirmed that there is a limited correlation between the level of serum BTMs and BMD changes [10,11]. Regretfully, few convenient and accurate biomarkers are currently available for diagnosis in the clinic.

MicroRNA (miRNA) is a type of small noncoding single-stranded RNA with 18 to 24 nucleotides [12]. As one of the epigenetic mechanisms regulating gene expression, miRNAs mediate the posttranscriptional gene-silencing of their target genes [13]. The potential value of miRNAs as novel biomarkers for early diagnosis, treatment, and prognosis monitoring has been well verified in diseases such as cancer, obesity, and diabetes [12,14]. Although several studies have found aberrant miRNA expression in osteoporosis-induced cells and animal models [15], owing to the complexity of the pathogenesis of PMOP in humans and the imperfect public gene expression databases, the mechanisms underlying disease occurrence and progression remain to be fully elucidated. Further study on the abundance difference of miRNAs in circulating serum under pathological conditions may provide a new method to reflect the overall state of bone metabolism and the dynamic process of BMD change. Meanwhile, changes in certain miRNAs may even carry specific information about the source tissue [16] to realize the precise diagnosis and treatment of PMOP.

The primary aim of the study was to screen differentially expressed miRNAs (DEmiRNAs) in the serum of PMOP patients and postmenopausal-without-osteoporosis (n-PMOP) controls and to validate the feasibility of using key miRNAs as biomarkers for the clinical diagnosis of disease. As a secondary aim, this study explored the expression characteristics of key miRNAs in populations with different BMDs and at different body sites. In addition, the key target gene functions and signaling pathways related to DEmiRNAs that may be involved in PMOP onset and progression were also annotated. This study highlights a novel approach in PMOP diagnosis and provides new insights into the epigenetic regulatory mechanisms of the disease.

## 2. Materials and Methods

### 2.1. Participants

This study surveyed 795 community-dwelling, middle-aged and elderly female participants who were recruited from June 2020 to August 2021 at the First Affiliated Hospital of Sun Yat-sen University, Guangzhou, China. The inclusion criteria were as follows: (1) age ≥ 50 years; (2) menopausal duration ≥ 1 year; and (3) signed an informed consent form before study entry. The exclusion criteria were as follows: (1) any comorbidity that could significantly affect bone metabolism, e.g., thyroid disease, diabetes, cancer, kidney disease, or ankylosing spondylitis; (2) previous treatment with anti-osteoporosis drugs or hormones (vitamin D or/and calcium supplements were allowed), e.g., estrogen or glucocorticoids; and (3) a history of tobacco smoking or alcohol dependence within the last year. Finally, a total of 83 unrelated ethnic Han Chinese women were eligible and included in the analysis.

### 2.2. Study Design

**Figure 1:**
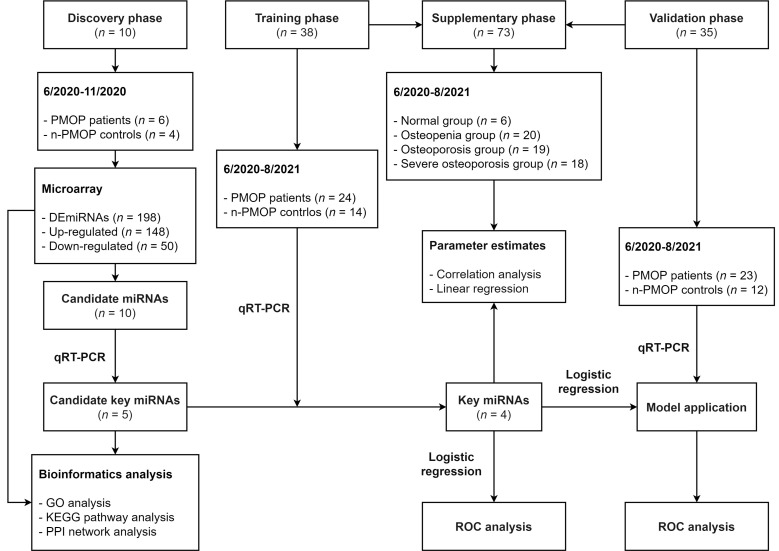
Flowchart of the study. PMOP, postmenopausal osteoporosis; n-PMOP, postmenopausal without osteoporosis; DEmiRNAs, differentially expressed miRNAs; qRT–PCR, quantitative real-time PCR; GO, Gene Ontology; KEGG, Kyoto Encyclopedia of Genes and Genomes; PPI, protein–protein interaction; and ROC, receiver operating characteristic.

The entire study was divided into 4 phases: discovery phase, training phase, validation phase, and supplementary phase (Figure 1). In the first 3 phases, participants were grouped into 2 categories: PMOP patients whose BMD T-score ≤ −2.5 and n-PMOP controls whose T-score > −2.5. In the supplementary phase, to analyze the relationship between key miRNAs and BMD, participants were divided into 4 groups [17]: (1) normal group (T-score ≥ −1); (2) osteopenia group (−2.5 < T-score < −1); (3) osteoporosis group (T-score ≤ −2.5); and (4) severe osteoporosis group (T-score ≤ −2.5 with fragility fracture).

All participants underwent BMD assessments on admission. Demographic and clinical data, including age, height, weight, age at menopause, and menopausal duration, were obtained, and body mass index (BMI) was calculated. The following serum BTMs and biochemical indices were also measured: (1) 25-hydroxy vitamin D (25[OH]D); (2) N-terminal middle segment osteocalcin (N-MID); (3) propeptide of type I procollagen (P1NP); (4) β-C-terminal telopeptide of type I collagen (β-CTX); (5) uric acid (UA); (6) alkaline phosphatase (ALP); (7) calcium; and (8) phosphorus.

This study was approved by the Ethics Committee of the First Affiliated Hospital of Sun Yat-sen University (Approval number: [2020]291–2).

### 2.3. Anthropometric Measurements

Participants wore lightweight clothing and removed their shoes before anthropometric assessments. Height and weight were measured by the corrected mechanical weight and height scale (RGZ-120, Suhong Medical Instruments Co., Changzhou, China), with an accuracy of 0.1 cm in height and 0.1 kg in weight. The average value from 3 measurements was taken for final evaluation.

Areal BMD was measured via DXA (Lunar iDXA, GE Healthcare, Chicago, IL, USA) of the lumbar spine (LS) 1–4, total hip (TH), and femoral neck (FN). All evaluations were performed by experienced diagnostic imaging physicians. The device was calibrated daily against a standard calibration phantom according to the manufacturer’s instructions. Based on prior measurements, the coefficient of variation (CV) for adult measurements is 0.8% for the LS, 0.8% for the FN, and 1.4% for the TH [10].

### 2.4. Biochemical and Immunological Analysis

Blood samples were collected via venipuncture, with participants having fasted overnight for at least 8 h. Whole blood was left to stand at room temperature for 30 min, and serum was then collected following centrifugation at 1200× *g* for 10 min at 4 °C.

The analyzers were calibrated daily before the analysis of all serum samples according to the manufacturer’s protocol. The routine clinical chemistry panel, including UA, ALP, calcium, and phosphorus, was detected using an AU5800 automatic biochemistry analyzer and its corresponding reagents (Beckman Coulter, Brea, CA, USA), with intra- and interassay CVs ranging from 0.5% to 4.9%. The special clinical immunology panel, including 25(OH)D, N-MID, P1NP and β-CTX, was measured using a Cobas 6000 analyzer series and its corresponding reagents (Roche, Basel, Switzerland, CH), with intra- and interassay CVs ranging from 0.6% to 4.3%.

### 2.5. RNA Extraction

Total RNA was extracted from 250 μL serum using TRlzol LS reagent (Invitrogen, Life Technologies, Carlsbad, CA, USA) according to the manufacturer’s protocol. The RNA quantity and quality were determined using an ND-1000 Spectrophotometer (NanoDrop Technologies, Wilmington, DE, USA).

### 2.6. Microarray

miRNA microarray analysis was performed by a commercial service (Kangcheng Biotech Co., Shanghai, China). Briefly, miRNA expression profiling was performed using an Agilent Human miRNA Microarray system, 8 × 60 K array (Agilent Technologies, Santa Clara, CA, USA), containing probes for 2549 human miRNAs based on the miRBase database (http://www.mirbase.org, accessed on 1 December 2020, version 21.0). RNA labeling and hybridization on the Agilent miRNA microarray chips were performed with an Agilent Quick Amp Labeling Kit (Agilent part number [p/n]: 5190-0442) and Agilent Gene Expression Hybridization Kit (Agilent p/n: 5188-5242). The hybridization images were captured with an Agilent Microarray Scanner (Agilent p/n: G2565BA) and digitized using Agilent Feature Extraction (version 11.0.1.1).

The microarray data in this study have been deposited in the Gene Expression Omnibus (GEO) database (http://www.ncbi.nlm.nih.gov/geo/, accessed on 1 May 2022; Accession number: GSE201543).

### 2.7. qRT–PCR

To confirm the findings obtained by analyzing the miRNA profiles, qRT–PCR analysis was performed using a QuantStudio5 Real-time PCR System (Applied Biosystems, Waltham, CA, USA). cDNA was obtained from 150 ng of total RNA using M-MuLV Reverse Transcriptase (Enzymatics p/n, P7040L). The PCR amplification procedures were performed according to a previous description and repeated in triplicate [18]. The relative expression level of miRNAs was normalized to that of the internal control hsa-miR-425-5p using the 2^−ΔΔCt^ cycle threshold method [19]. The primer sequences for the qRT–PCR assays are listed in Supplementary Table S1.

### 2.8. Bioinformatics Analysis

Three online databases, TargetScan (http://www.targetscan.org/vert_71/, accessed on 6 March 2021), miRTarBase (http://mirtarbase.mbc.nctu.edu.tw/php/index.php, accessed on 6 March 2021), and miRDB (http://mirdb.org/, accessed on 6 March 2021), were used to predict the potential target genes of DEmiRNAs. Gene Ontology (GO) analysis and Kyoto Encyclopedia of Genes and Genomes (KEGG) enrichment analysis were performed using the R package (version 3.6.0) “clusterProfiler” to evaluate the biological functions and pathways associated with target genes. The STRING online database (http://string-db.org/, accessed on 7 March 2021, version 11.0) was used to construct a protein–protein interaction (PPI) network and select hub genes. The minimum interaction score was set as 0.9. Cytoscape (http://cytoscape.org/, accessed on 7 March 2021, version 3.8.0) was used to visualize the PPI network.

### 2.9. Statistical Analysis

Statistical analyses were performed using IBM SPSS Statistics (IBM, Armonk, NY, USA; version 22.0). Data are presented as the mean ± standard deviation. Independent sample t-tests and one-way analysis of variance (ANOVA) were performed to analyze the data. Binary logistic regression analyses were performed to calculate the predicted probability of different diagnostic combinations. Receiver operating characteristic (ROC) curves were plotted to evaluate the diagnostic effects of the models. The area under the ROC curve (AUC) for different prediction models was compared using the method described by DeLong et al. [20]. To investigate associations between key miRNAs and the BMD at different body sites, Pearson’s correlation and partial correlation analyses were used. Multiple linear regression models were conducted to examine the factors that influenced changes in BMD. The variance inflation factor (VIF) was calculated to assess the collinearity of independent variables, and an independent variable with VIF > 10 was considered highly collinear. Differences were considered statistically significant at *p* < 0.05.

## 3. Results

### 3.1. Clinical Characteristics of the Participants

The characteristics of the participants in the discovery set, training set, and validation set are shown in Table 1. In the discovery set and training set, no significant differences in participant characteristics, including age, BMI, age at menopause, or menopausal duration, were observed between the PMOP patients and n-PMOP controls (*p* > 0.05). In the validation set, age and menopausal duration were significantly higher in the 23 PMOP patients than in the 12 n-PMOP controls (67.5 ± 8.8 vs. 58.3 ± 8.1, *p* = 0.005; 16.0 ± 8.5 vs. 6.3 ± 6.1, *p* = 0.001, respectively). The differences in BTMs and biochemical indices did not reach statistical significance between PMOP patients and n-PMOP controls in the discovery set, training set, or validation set (*p* > 0.05), except for calcium in the discovery set (2.10 ± 0.13 vs. 2.35 ± 0.06, *p* = 0.006).

### 3.2. Screening of Key miRNAs

**Figure 2:**
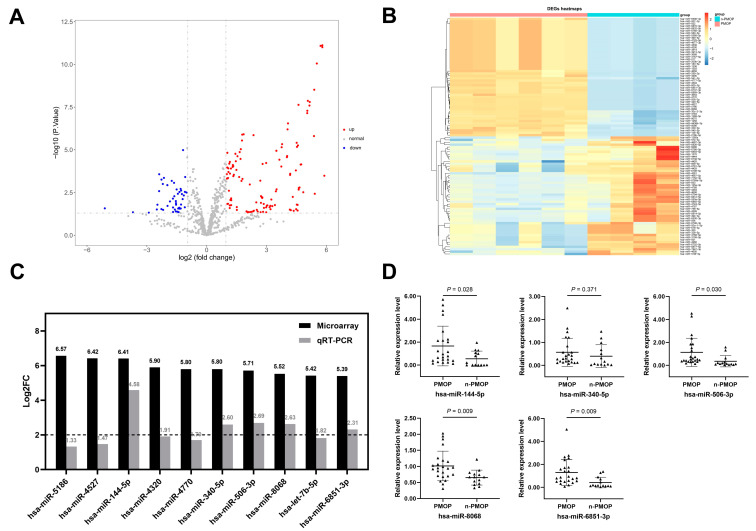
Screening of key miRNAs. (**A**) Volcano plot for visualization of DEmiRNAs. (**B**) Heatmap for visualization of the top 50 upregulated and downregulated DEmiRNAs. (**C**) Five candidate key miRNAs were obtained according to the screening criteria |Log2FC| ≥ 2 and *p* < 0.05. (**D**) Relative expression levels of candidate key miRNAs in the training set. DEmiRNAs, differentially expressed miRNAs.

The expression levels of 2549 miRNAs were measured in the discovery set. Under the criteria of adj. *p* < 0.05 and |Log2 Fold Change (FC)| > 1, a total of 198 DEmiRNAs were screened (Supplementary Table S2). Compared with the n-PMOP controls, 148 miRNAs were significantly upregulated and 50 miRNAs were significantly downregulated in the PMOP patients. A volcano plot was constructed to demonstrate the profiles of the DEmiRNAs (Figure 2A), and the 50 most upregulated and downregulated miRNAs are shown in the heatmap (Figure 2B). Among the DEmiRNAs, ten miRNAs with the highest FC in expression levels compared with the n-PMOP controls were selected as candidates for verification by qRT–PCR. The qRT–PCR results showed that the differential expression of candidate miRNAs was generally consistent with the microarray results (Supplementary Table S3).

To further expand the differences in candidate miRNA expression, the screening criteria |Log2FC| ≥ 2 and *p* < 0.05 were applied, and five candidate key miRNAs were obtained, namely, hsa-miR-144-5p, hsa-miR-340-5p, hsa-miR-506-3p, hsa-miR-8068, and hsa-miR-6851-3p (Figure 2C). Subsequently, we further evaluated the candidate key miRNAs in an independent training cohort containing 24 PMOP patients and 14 n-PMOP controls (Supplementary Table S4), and the results are shown in Figure 2D. Except for hsa-miR-340-5p (*p* = 0.371), the relative expression levels of the other four candidate key miRNAs were significantly different between groups (*p* < 0.05). Therefore, the study finally included hsa-miR-144-5p, hsa-miR-506-3p, hsa-miR-8068, and hsa-miR-6851-3p as key miRNAs in the subsequent model construction and verification.

### 3.3. Diagnostic Performance of DEmiRNAs

**Figure 3:**
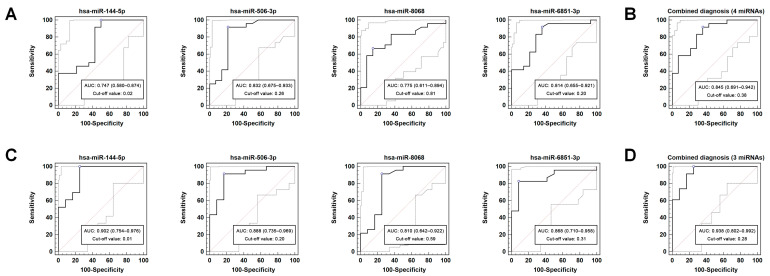
ROC curve of key miRNAs for PMOP diagnosis. (**A**) Independent diagnostic performance of individual miRNAs in the training set. (**B**) Combined diagnosis using 4 key miRNAs showed the highest accuracy in the training set. (**C**) Independent diagnostic performance of individual miRNAs in the validation set. (**D**) Combined diagnosis using 3 key miRNAs (hsa-miR-144-5p, hsa-miR-506-3p, and hsa-miR-6851-3p) showed the highest accuracy in the validation set. ROC, receiver operating characteristic; PMOP, postmenopausal osteoporosis; and AUC, area under the curve.

To identify potential miRNAs for diagnostic model construction, the performance of each key miRNA for PMOP detection was first investigated. Subsequently, logistic regression was used to build combined diagnostic models that could explore whether combinations of biomarkers could improve the diagnostic efficacy. We calculated the AUC, sensitivity, specificity, positive predictive value (PPV), and negative predictive value (NPV) of the models to find the optimal diagnostic combination. Supplementary Table S5 summarizes the diagnostic performance of the models in the training set. All four key miRNAs showed acceptable independent diagnostic efficacy (AUC range: 0.747–0.832; Figure 3A), and combination of the four key miRNAs had the greatest diagnostic potential, with the highest AUC (AUC: 0.845, 95% confidence interval (CI): 0.691–0.942; sensitivity: 91.7%, 95% CI: 73.0–99.0%; specificity: 64.3%, 95% CI: 35.1–87.2%; PPV, 81.5%, 95% CI: 61.9–93.7%; and NPV, 81.8%, 95% CI: 48.2–97.7%; Figure 3B).

In the validation set, the significant difference in the relative expression levels of the four key miRNAs between the PMOP patients and the n-PMOP controls was further confirmed (Supplementary Figure S1). Applying the same model to the validation set (Table 2), the independent diagnostic performance of all four key miRNAs was improved (Figure 3C). The combined diagnostic model composed of hsa-miR-144-5p, hsa-miR-506-3p, and hsa-miR-6851-3p achieved the highest diagnostic value (AUC: 0.938, 95% CI: 0.802–0.992; sensitivity: 100.0%, 95% CI: 85.2–100.0%; specificity: 75.0%, 95% CI: 42.8–94.5%; PPV, 88.5%, 95% CI: 69.8–97.6%; NPV, 100.0%, 95% CI: 66.4–100.0%; Figure 3D), higher than the diagnostic model composed of the four key miRNAs in the training set.

Using the method of Delong et al. [20], the differences in the AUC among different diagnostic models were examined in the training set and validation set. The results showed that although the combination of multiple miRNAs improved the AUC to a certain extent, there was no significant difference between these AUCs in either the training set or validation set (*p* > 0.05).

### 3.4. Relative Expression Levels of Key miRNAs in Different Clinical Stages

Seventy-three participants in the training set and validation set were divided into four groups based on specific guidelines [17], and the demographics data and serum indices are shown in Supplementary Table S6. The age and menopausal duration in the severe osteoporosis group (68.7 ± 5.5 and 16.2 ± 6.5, respectively) were significantly higher than in the normal group (59.2 ± 6.3 and 5.7 ± 4.5, respectively) and osteopenia group (61.4 ± 7.8 and 10.0 ± 7.0, respectively) (*p* < 0.05). However, no significant difference was observed between the severe osteoporosis group and the osteoporosis group in terms of demographics (*p* > 0.05). ALP levels (108.2 ± 36.3) in the serum of the severe osteoporosis group were the highest among the four groups (*p* < 0.05). N-MID, ALP, and phosphorus were the only three serum indices that showed significant differences among the four groups (*p* < 0.05).

**Figure 4:**
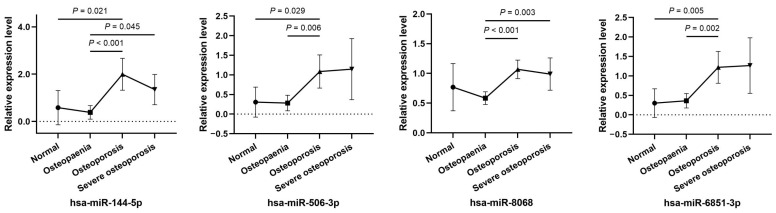
Comparison of the relative expression levels of key miRNAs among the 4 groups.

The relative expression levels of the key miRNAs in serum are shown in Figure 4. hsa-miR-144-5p, hsa-miR-506-3p, and hsa-miR-6851-3p were highly expressed in the osteoporosis group, and the differences were significant compared with the normal group and osteopenia group (*p* < 0.05). In addition, hsa-miR-144-5p and hsa-miR-8068 were expressed at lower levels in the osteopenia group than in the severe osteoporosis group (*p* < 0.05). Differences in key miRNA serum levels among subgroups may partly reflect the unique bone metabolic patterns in various stages of disease.

### 3.5. Correlations between Key miRNAs and BMD

According to the results of Pearson correlation analysis, hsa-miR-144-5p was significantly negatively correlated with FN BMD (*p* < 0.05), but there was no significant correlation with the BMD at other body sites (*p* > 0.05); hsa-miR-506-3p and hsa-miR-8068 were significantly associated with LS 1–4 BMD (*p* < 0.05), while hsa-miR-6851-3p was significantly correlated with BMD at all three body sites (*p* < 0.05). Additionally, age and menopausal duration were used as covariates for partial correlation analysis. Apart from hsa-miR-8068, the other candidate key miRNAs had different degrees of negative correlations with BMD at LS 1–4, TH, and FN (*p* < 0.05), and the correlations were further enhanced (Table 3).

To further evaluate the influencing factors of the key miRNAs on BMD at different body sites, multivariable linear regression was performed using the key miRNAs as independent variables and BMD as a dependent variable. Meanwhile, variables that were significant in univariate analyses were adopted as covariates. No multicollinearity was detected among the independent variables in the regression models. Table 4 shows the results from the linear regression models. In Model 1, only hsa-miR-6851-3p was found to be significantly negatively associated with LS 1–4 BMD (*β* = −0.851, *p* = 0.007). When controlling for age, menopausal duration, N-MID, ALP, and phosphorus as covariates in Model 2, hsa-miR-6851-3p remained a strong predictor of LS 1–4 BMD (*β* = −0.645, *p* = 0.026), while hsa-miR-144-5p was determined to be the lone predictor to have significant predictive power for BMD at different body sites (LS 1–4: *β* = −0.265, *p* = 0.022; TH: *β* = −0.301, *p* = 0.005; and FN: *β* = −0.324, *p* = 0.003, respectively). No significant correlation was found between BMD and hsa-miR-506-3p or hsa-miR-8068 (*p* > 0.05).

### 3.6. Target Genes and Pathways Correlated with DEmiRNAs

**Figure 5:**
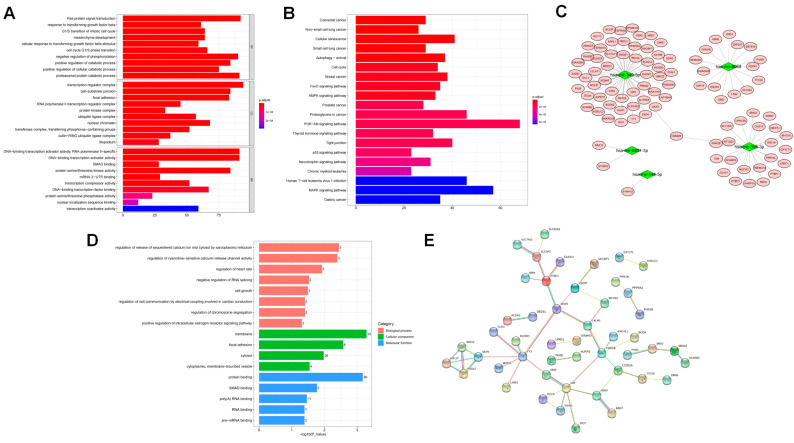
Bioinformatics analysis based on DEmiRNA target genes. (**A**) The top 10 significant terms in GO analysis (BP/CC/MF). (**B**) The top 20 significant terms in KEGG enrichment analysis. (**C**) The regulatory network of candidate key miRNA target genes. (**D**) GO analysis of targeted genes of candidate key miRNAs. (**E**) PPI network of candidate key miRNA target genes. DEmiRNAs, differentially expressed miRNAs; GO, Gene Ontology; BP, biological process; CC, cellular component; MF, molecular function; KEGG, Kyoto Encyclopedia of Genes and Genomes; and PPI, protein–protein interaction.

On the basis of the 198 DEmiRNAs, 1945 target genes were identified using the TargetScan, miRTarBase, and miRDB databases. The top 10 miRNAs and their target genes are shown in Supplementary Table S7. In total, 1120 GO terms and 85 KEGG pathways were enriched in target genes according to the criteria adj. *p* < 0.05 (Supplementary Tables S8 and S9). The top 10 enriched terms of GO and KEGG pathways are shown in Figure 5A,B. The 1945 target genes were imported into the STRING database to construct a PPI network. After excluding the isolated nodes, the final PPI network is composed of 1052 nodes and 7477 edges, as is shown in Supplementary Figure S2. The top 20 hub genes were sorted by degree (refers to the number of gene connections within the network) in descending order (Supplementary Table S10).

Ninety-four target genes for the five candidate key miRNAs were also predicted. As shown in Figure 5C, hsa-miR-340-5p and hsa-miR-144-5p cooperatively regulate the target gene *CREBRF*. *SYNPO2* is the only predicted target gene of hsa-miR-144-5p. The function of target genes was significantly enriched in 17 GO terms (Biological Process [BP]:Cellular Component [CC]:Molecular Function [MF] = 8:4:5) and a KEGG signaling pathway (“neurotrophin signaling pathway”). For the BP classification, the top three enrichment terms were “regulation of release of sequestered calcium ion into cytosol by sarcoplasmic reticulum”, “regulation of ryanodine-sensitive calcium-release channel activity”, and “regulation of heart rate”. Membrane, focal adhesion, and cytosol were the most highly enriched CC terms. In the MF category, “protein binding”, “Smad binding”, and “poly(A) RNA binding” were significantly enriched (Figure 5D). For visualization, the PPI network of target genes is presented in Figure 5E. *YY1*, *VIM*, and *YWHAE* were the top three hub genes, with higher interaction levels (Table 5).

## 4. Discussion

Since the first discovery of miRNAs in Caenorhabditis elegans in 1993, these molecules have been widely confirmed to exist in more than 12 types of mammalian body fluids, including serum [12,21]. The high degree of conservation [22], detectability [23], and specific spatiotemporal expression [14] of serum miRNAs make them promising biomarkers for liquid biopsy. As important regulators of gene expression, an increasing number of studies have confirmed that miRNAs are involved in regulating the pathological progression of PMOP [24,25,26]. However, because of the absence of clinical validation, none of them has been widely recommended for clinical diagnostic purposes. In this study, four key miRNAs were screened and validated in independent populations. The results revealed that hsa-miR-144-5p, hsa-miR-506-3p, hsa-miR-8068, and hsa-miR-6851-3p are potential independent biomarkers for distinguishing PMOP patients from n-PMOP controls. This finding provides additional information for PMOP diagnosis, independent of radiological findings and BTMs.

Several small clinical studies have preliminarily explored the potential of abnormally expressed circulating miRNAs as diagnostic biomarkers of PMOP [27,28,29]. For example, miR-133a, a stimulator of osteoclastogenesis, is useful for the detection of PMOP [29]. However, the results of previous studies are inconsistent, possibly due to the different populations studied. Moreover, the diagnostic accuracy and clinical usability of certain miRNAs for disease in certain clinical settings are limited. A study by Mandourah et al. reported that hsa-miR-122-5p and hsa-miR-4516 were downregulated in blood samples and could serve as potential diagnostic markers of osteoporosis, but the diagnostic values are not yet sufficient (AUC = 0.752) [28]. In our study, four key miRNAs showed high diagnostic value, with AUCs greater than 0.7 in both the training set and validation set, and the AUCs using the combined miRNAs for diagnosis were higher than 0.9.

Currently, the functions of hsa-miR-144-5p and hsa-miR-506-3p have been reported mainly in the cancer field. High plasma levels of hsa-miR-144-5p were shown to be associated with renal cell carcinoma [30], non-small-cell lung cancer [31] or glioblastoma [32]. Recent research by Zhang et al. showed that miR-144-5p can reduce bone repair and regeneration in type 2 diabetes by suppressing the expression of Smad1 [33]. In this study, linear regression analysis revealed that hsa-miR-144-5p was independently associated with BMD changes in multiple body sites, and the association between its expression pattern and PMOP progression is promising for further longitudinal research. Overexpression of hsa-miR-506-3p was found to inhibit the proliferation, migration, and invasion of cancer cells in osteosarcoma [34], prostate cancer [35], and hepatocellular carcinoma [36]. Thus far, the function and serum expression profiles of hsa-miR-8068 and hsa-miR-6851-3p have not been extensively investigated. The results of the present study demonstrate for the first time the additional values of the above miRNAs in PMOP.

The predicted expression profiles of the target genes confirmed the close association between DEmiRNAs and bone metabolism. As reported by Jeong et al., *YY1* significantly inhibited Runx2-mediated transcriptional activity of osteocalcin (OCN) and ALP promoters, and knockout of this gene enhanced the osteoblast differentiation induced by *BMP2* and *Runx2* [37]. At the same time, *YY1* can modulate the transcriptional activity of *Smad*, thereby regulating cell differentiation induced by the transforming growth factor superfamily signaling pathway [38]. VIM is a type III intermediate filament protein that is expressed in mesenchymal cells [39]. Overexpression of VIM in osteoblasts inhibits osteoblast differentiation, as shown by reduced ALP activity, delayed mineralization, and reduced expression of osteoblast markers [40]. This effect may be mediated by VIM competitively binding ATF4, which is required for *OCN* transcription and osteoblast differentiation [41]. YWHAE belongs to the 14-3-3 protein family and is involved in the transduction of signaling pathways by binding to phosphoserine-containing proteins [42]. A study on YWHAE in exosomes released from an osteoblast/osteocyte coculture system revealed that YWHAE had a positive response to mechanical stress [43]. Rivero et al. loaded 14-3-3ε protein into a scaffold, and positive stimulation of osteogenicity was observed [44]. These reports regarding the functions of target genes predicted by DEmiRNA profiles support our finding that elevated levels of differentially expressed serum miRNAs are correlated with PMOP.

Despite its strengths as discussed above, this research had certain limitations. First, we used strict inclusion criteria to obtain relatively homogenous populations. However, a power calculation was not performed a priori, and the small sample size may have resulted in decreased statistical power. Second, despite accounting for many important confounders, we cannot exclude residual confounding by unmeasured or unknown confounders. Thus, these findings need to be validated in subsequent large sample studies. In addition, further functional validation in vitro and in vivo is required to confirm the associations between these molecules and disease.

## 5. Conclusions

This study presents new clinical evidence regarding the deregulated expression of miRNAs in the serum of PMOP patients. Our results indicate that hsa-miR-144-5p, hsa-miR-506-3p, hsa-miR-8068, and hsa-miR-6851-3p target a variety of bone-metabolism-related genes and pathways and are potential independent biomarkers for clinical diagnosis of the disease, outperforming traditional BTMs. Among them, hsa-miR-144-5p is the only key miRNA that can simultaneously predict changes in BMD in LS 1–4, TH, and FN. These findings provide not only a new method for clinicians to evaluate the changes in BMD in postmenopausal women under limited conditions but also a meaningful inspiration for in-depth study of the epigenetic regulation mechanisms underlying PMOP onset and progression.

## Figures and Tables

**Table 1:** General clinical data, serum BTMs, and biochemical indices of participants.

Variable	Reference Range	Discovery Set (*n* = 10)			Training Set (*n* = 38)			Validation Set (*n* = 35)		
		PMOP (*n* = 6)	n-PMOP (*n* = 4)	*p*	PMOP (*n* = 24)	n-PMOP (*n* = 14)	*p*	PMOP (*n* = 23)	n-PMOP (*n* = 12)	*p*
Age (years)	-	74.3 ± 6.8	70.0 ± 8.2	0.387	66.5 ± 7.6	63.1 ± 6.3	0.158	67.5 ± 8.8	58.3 ± 8.1	0.005
BMI (g/cm^2^)	-	24.8 ± 3.6	25.2 ± 2.8	0.850	22.7 ± 3.7	24.2 ± 2.7	0.181	21.9 ± 3.1	23.6 ± 2.6	0.113
Age at menopause (years)	-	52.3 ± 2.1	52.8 ± 1.9	0.755	51.8 ± 3.8	51.9 ± 4.0	0.985	51.4 ± 3.7	52.1 ± 3.2	0.588
Menopausal duration (years)	-	22.0 ± 6.1	17.3 ± 8.5	0.331	14.8 ± 7.8	11.2 ± 6.5	0.162	16.0 ± 8.5	6.3 ± 6.1	0.001
LS 1–4 BMD (T-score)	-	−2.2 ± 0.7	−1.1 ± 1.4	0.121	−2.9 ± 1.2	−1.2 ± 1.0	<0.001	−3.0 ± 1.1	−0.8 ± 0.7	<0.001
TH BMD (T-score)	-	−2.1 ± 0.3	−1.5 ± 0.6	0.078	−2.3 ± 0.7	−1.3 ± 0.9	0.001	−2.6 ± 0.9	−1.0 ± 0.7	<0.001
FN BMD (T-score)	-	−2.7 ± 0.5	−1.9 ± 0.4	0.035	−2.7 ± 0.6	−1.5 ± 0.8	<0.001	−2.9 ± 0.7	−1.5 ± 0.7	<0.001
LS 1–4 BMD (g/cm^2^)	-	0.843 ± 0.072	1.010 ± 0.219	0.113	0.760 ± 0.135	0.970 ± 0.124	<0.001	0.757 ± 0.128	0.992 ± 0.097	<0.001
TH BMD (g/cm^2^)	-	0.699 ± 0.042	0.790 ± 0.066	0.027	0.685 ± 0.097	0.808 ± 0.121	0.001	0.639 ± 0.109	0.850 ± 0.090	<0.001
FN BMD (g/cm^2^)	-	0.610 ± 0.057	0.717 ± 0.035	0.010	0.617 ± 0.069	0.746 ± 0.093	<0.001	0.586 ± 0.086	0.751 ± 0.081	<0.001
25(OH)D (ng/mL)	>25	29.7 ± 10.2	29.3 ± 5.9	0.944	23.1 ± 7.5	22.6 ± 8.6	0.836	24.4 ± 9.2	26.3 ± 4.8	0.509
N-MID (ng/mL)	14–46	22.47 ± 4.63	21.25 ± 9.23	0.785	20.30 ± 8.73	16.36 ± 6.96	0.158	17.87 ± 9.13	19.12 ± 5.58	0.667
P1NP (ng/mL)	0.00–36.40	53.50 ± 17.17	34.33 ± 9.26	0.078	49.01 ± 20.15	49.37 ± 31.90	0.967	57.20 ± 35.58	67.29 ± 31.51	0.415
β-CTX (ng/mL)	≤1.008	0.397 ± 0.135	0.338 ± 0.057	0.368	0.618 ± 0.467	0.438 ± 0.268	0.197	0.616 ± 0.538	0.621 ± 0.248	0.978
UA (μmol/L)	140–360	334.2 ± 76.6	330.3 ± 88.7	0.942	297.6 ± 89.8	299.0 ± 64.5	0.960	306.7 ± 68.7	303.5 ± 113.9	0.917
ALP (U/L)	0–110	76.5 ± 12.6	72.5 ± 8.2	0.593	84.3 ± 35.2	73.3 ± 36.8	0.367	90.5 ± 29.6	74.7 ± 16.0	0.095
Calcium (mmol/L)	2.10–2.60	2.10 ± 0.13	2.35 ± 0.06	0.006	2.28 ± 0.16	2.24 ± 0.19	0.508	2.28 ± 0.16	2.22 ± 0.17	0.301
Phosphorus (mmol/L)	0.97–1.62	1.12 ± 0.17	1.23 ± 0.21	0.398	1.16 ± 0.21	1.14 ± 0.19	0.699	1.17 ± 0.16	1.18 ± 0.19	0.796

Note: Data are presented as mean ± standard deviation. All *p*-values were calculated with the *t*-test. *p*-value < 0.05 was considered to indicate a statistically significant difference. BTMs, bone turnover markers; BMI, body mass index; LS, lumbar spine; TH, total hip; FN, femoral neck; 25(OH)D, 25-hydroxy vitamin D; N-MID, N-terminal middle segment osteocalcin; P1NP, propeptide of type I procollagen; β-CTX, β-C-terminal telopeptide of type I collagen; UA, uric acid; and ALP, alkaline phosphatase.

**Table 2:** Performance characteristics of the miRNAs for diagnosing PMOP (validation set).

Variable	AUC (95% CI)	*p*	Sensitivity (95% CI)	Specificity (95% CI)	PPV (95% CI)	NPV (95% CI)
Numbers of miRNA: 1						
hsa-miR-144-5p	0.902 (0.754–0.976)	<0.001	100.0 (85.2–100.0)	75.0 (42.8–94.5)	88.5 (69.8–97.6)	100.0 (66.4–100.0)
hsa-miR-506-3p	0.888 (0.735–0.969)	<0.001	91.3 (72.0–98.9)	83.3 (51.6–97.9)	91.3 (72.0–98.9)	83.3 (51.6–97.9)
hsa-miR-8068	0.810 (0.642–0.922)	<0.001	91.3 (72.0–98.9)	75.0 (42.8–94.5)	87.5 (67.6–97.3)	81.8 (48.2–97.7)
hsa-miR-6851-3p	0.868 (0.710–0.958)	<0.001	82.6 (61.2–95.0)	91.7 (61.5–99.8)	95.0 (75.1–99.9)	73.3 (44.9–92.2)
Numbers of miRNA: 2						
hsa-miR-144-5p + hsa-miR-506-3p	0.917 (0.773–0.983)	<0.001	100.0 (85.2–100.0)	75.0 (42.8–94.5)	88.5 (69.8–97.6)	100.0 (66.4–100.0)
hsa-miR-144-5p + hsa-miR-8068	0.899 (0.749–0.975)	<0.001	100.0 (85.2–100.0)	75.0 (42.8–94.5)	88.5 (69.8–97.6)	100.0 (66.4–100.0)
hsa-miR-144-5p + hsa-miR-6851-3p	0.928 (0.787–0.988)	<0.001	91.3 (72.0–98.9)	83.3 (51.6–97.9)	91.3 (72.0–98.9)	83.3 (51.6–97.9)
hsa-miR-506-3p + hsa-miR-8068	0.917 (0.773–0.983)	<0.001	100.0 (85.2–100.0)	75.0 (42.8–94.5)	88.5 (69.8–97.6)	100.0 (66.4–100.0)
hsa-miR-506-3p + hsa-miR-6851-3p	0.891 (0.740–0.971)	<0.001	82.6 (61.2–95.0)	91.7 (61.5–99.8)	95.0 (75.1–99.9)	73.3 (44.9–92.2)
hsa-miR-8068 + hsa-miR-6851-3p	0.924 (0.782–0.986)	<0.001	95.7 (78.1–99.9)	75.0 (42.8–94.5)	88.0 (68.8–97.5)	90.0 (55.5–99.7)
Numbers of miRNA: 3						
hsa-miR-144-5p + hsa-miR-506-3p + hsa-miR-8068	0.917 (0.773–0.983)	<0.001	100.0 (85.2–100.0)	75.0 (42.8–94.5)	88.5 (69.8–97.6)	100.0 (66.4–100.0)
hsa-miR-144-5p + hsa-miR-506-3p + hsa-miR-6851-3p	0.938 (0.802–0.992)	<0.001	100.0 (85.2–100.0)	75.0 (42.8–94.5)	88.5 (69.8–97.6)	100.0 (66.4–100.0)
hsa-miR-506-3p + hsa-miR-8068 + hsa-miR-6851-3p	0.913 (0.768–0.981)	<0.001	95.7 (78.1–99.9)	75.0 (42.8–94.5)	88.0 (68.8–97.5)	90.0 (55.5–99.7)
hsa-miR-144-5p + hsa-miR-8068 + hsa-miR-6851-3p	0.935 (0.797–0.990)	<0.001	100.0 (85.2–100.0)	75.0 (42.8–94.5)	88.5 (69.8–97.6)	100.0 (66.4–100.0)
Numbers of miRNA: 4						
hsa-miR-144-5p + hsa-miR-506-3p + hsa-miR-8068 + hsa-miR-6851-3p	0.928 (0.787–0.988)	<0.001	100.0 (85.2–100.0)	75.0 (42.8–94.5)	88.5 (69.8–97.6)	100.0 (66.4–100.0)

Note: Data are presented as value, or value (95% CI). *p*-value < 0.05 was considered to indicate a statistically significant difference. PMOP, postmenopausal osteoporosis; AUC, area under the curve; CI, confidence interval; PPV, positive predictive value; and NPV, negative predictive value.

**Table 3:** Correlations between key miRNAs and BMD (g/cm^2^) at different body sites.

Variable	LS 1–4		TH		FN	
	Simple	Partial	Simple	Partial	Simple	Partial
hsa-miR-144-5p	−0.230	−0.311 **	−0.230	−0.343 **	−0.265 *	−0.391 **
hsa-miR-506-3p	−0.267 *	−0.316 **	−0.207	−0.261 *	−0.245 *	−0.310 **
hsa-miR-8068	−0.257 *	−0.263 *	−0.190	−0.181	−0.212	−0.207
hsa-miR-6851-3p	−0.357 **	−0.383 **	−0.248 *	−0.265 *	−0.276 *	−0.301 *

Note: Data are presented as Pearson correlation coefficient (*r*). All *p*-values were calculated with the Pearson correlation or covariate-adjusted partial correlation test. *p* < 0.05 was considered to indicate a statistically significant difference. * *p* < 0.05; ** *p* < 0.01. Covariates included age and menopausal duration. BMD, body mineral density; LS, lumbar spine; TH, total hip; and FN, femoral neck.

**Table 4:** Linear regression analysis of the factors influencing BMD (g/cm^2^) change.

Variable	LS 1–4					TH					FN				
	B (95% CI)	*β*	*t*	*p*	*R* ^2^	B (95% CI)	*β*	*t*	*p*	*R* ^2^	B (95% CI)	*β*	*t*	*p*	R^2^
Model 1															
hsa-miR-144-5p	−0.017 (−0.044, 0.010)	−0.156	−1.268	0.209	0.187	−0.016 (−0.039, 0.007)	−0.182	−1.405	0.165	0.096	−0.015 (−0.033, 0.003)	−0.207	−1.617	0.110	0.114
hsa-miR-506-3p	0.091 (−0.001, 0.183)	0.630	1.979	0.052		0.035 (−0.043, 0.114)	0.300	0.894	0.374		0.021 (−0.042, 0.083)	0.217	0.654	0.515	
hsa-miR-8068	−0.022 (−0.134, 0.090)	−0.061	−0.394	0.695		−0.006 (−0.102, 0.089)	−0.021	−0.128	0.898		−0.002 (−0.078, 0.075)	−0.006	−0.039	0.969	
hsa-miR-6851-3p	−0.128 (−0.220, −0.036)	−0.851	−2.774	0.007		−0.055 (−0.134, 0.024)	−0.451	−1.395	0.168		−0.040 (−0.103, 0.023)	−0.403	−1.259	0.212	
Model 2															
Age	0.011 (0.001, 0.020)	0.550	2.223	0.030	0.412	0.008 (0.001, 0.015)	0.498	2.161	0.035	0.490	0.006 (0.000, 0.012)	0.465	2.044	0.045	0.503
Menopausal duration	−0.016 (−0.026, −0.006)	−0.808	−3.272	0.002		−0.015 (−0.022, −0.008)	−0.928	−4.036	<0.001		−0.012 (−0.018, −0.006)	−0.933	−4.116	<0.001	
N-MID	0.002 (−0.002, 0.006)	0.098	0.921	0.360		0.002 (−0.001, 0.006)	0.149	1.499	0.139		0.001 (−0.001, 0.004)	0.087	0.889	0.377	
ALP	−0.001 (−0.002, 0.000)	−0.237	−2.236	0.029		−0.001 (-0.002, 0.000)	−0.220	−2.234	0.029		−0.001 (−0.001, 0.000)	−0.196	−2.016	0.048	
Phosphorus	0.030 (−0.154, 0.214)	0.034	0.326	0.746		0.114 (−0.025, 0.253)	0.160	1.638	0.106		0.075 (−0.036, 0.186)	0.130	1.345	0.183	
hsa-miR-144-5p	−0.029 (−0.054, −0.004)	−0.265	−2.357	0.022		−0.027 (−0.045, −0.008)	−0.301	−2.876	0.005		−0.023 (−0.038, −0.008)	−0.324	−3.131	0.003	
hsa-miR-506-3p	0.067 (−0.018, 0.152)	0.465	1.578	0.120		0.014 (−0.050, 0.078)	0.120	0.436	0.665		0.001 (−0.051, 0.052)	0.007	0.024	0.981	
hsa-miR-8068	0.000 (−0.100, 0.101)	0.001	0.006	0.995		0.021 (−0.056, 0.097)	0.070	0.538	0.592		0.021 (−0.040, 0.082)	0.087	0.681	0.499	
hsa-miR-6851-3p	−0.097 (−0.182, −0.012)	−0.645	−2.275	0.026		−0.031 (−0.096, 0.033)	−0.256	−0.968	0.337		−0.017 (−0.069, 0.034)	−0.177	−0.678	0.500	

Note: Data are presented as value, or value (95% CI). *p*-value < 0.05 was considered to indicate a statistically significant difference. Dependent variables were LS1–4 BMD; covariates in Model 2 were age, menopausal duration, N-MID, ALP, and Phosphorus. BMD, bone mineral density; LS, lumbar spine; TH, total hip; FN, femoral neck; B, non-standardized regression coefficient; CI, confidence interval; *β*, standardized regression coefficient; *R*^2^, coefficient of determination; N-MID, N-terminal middle segment osteocalcin; and ALP, alkaline phosphatase.

**Table 5:** The top 14 target genes and their degree of enrichment (5 candidate key miRNAs).

Gene	Degree	Gene	Degree
*YY1*	7	*UBE2Z*	3
*VIM*	6	*MYLIP*	3
*YWHAE*	5	*FBXL3*	3
*SKP2*	4	*ZWINT*	3
*PTBP1*	4	*NEK9*	3
*DDX5*	4	*CC2D2A*	3
*SLC3A2*	3	*CALM1*	3

## Data Availability

The datasets supporting the conclusions of this article are included within the paper and its Supplementary Materials. All other datasets used and analyzed during the study are available from the corresponding author on reasonable request.

## Supplementary Materials

The following supporting information can be downloaded at: https://www.mdpi.com/article/10.3390/diagnostics12112872/s1, Supplementary Figure S1: Relative expression levels of key miRNAs in the validation set; Supplementary Figure S2: PPI network of DEmiRNA target genes; Supplementary Table S1: Primer sequences used for qRT–PCR; Supplementary Table S2: Upregulated and downregulated miRNAs between PMOP patients and n-PMOP controls; Supplementary Table S3: Verification of microarray results by qRT–PCR; Supplementary Table S4: qRT–PCR results for candidate key miRNAs in the training set; Supplementary Table S5: Performance characteristics of the miRNAs for diagnosing PMOP (training set); Supplementary Table S6: General clinical data, serum BTMs, and biochemical indices of participants with different BMDs; Supplementary Table S7: The top 10 upregulated and downregulated DEmiRNAs and their target genes; Supplementary Table S8: GO analysis of DEmiRNA target genes; Supplementary Table S9: KEGG enrichment analysis of DEmiRNA target genes; Supplementary Table S10: The top 20 target genes and their degree of enrichment (DEmiRNAs).
