# Supplementary material for: Bone-Metabolism-Related Serum microRNAs to Diagnose Osteoporosis in Middle-Aged and Elderly Women
Source: Diagnostics (Basel). 2022 Nov 19;12(11):2872. doi: 10.3390/diagnostics12112872 (PMC9689310; doi:10.3390/diagnostics12112872)
Supplement: Supplementary file 1 [file diagnostics-12-02872-s001.zip › Supplementary Table S1.pdf]

Supplemental Table S1. Primer sequences used for qRT–PCR

Gene	Primer	Annealing temperature (°C)	Product length (bp)
hsa-miR-5186	GSP:5'GGGGGGAGAGATTGGTAGAAA3'<br>R:5'GTGCGTGTCGTGGAGTCG3'	60	65
hsa-miR-4527	GSP:5'GGGATGGTCTGCAAAGAGAT3'<br>R:5'GTGCGTGTCGTGGAGTCG3'	60	64
hsa-miR-144-5p	GSP:5'GGGGGGGATATCATCATATAC3'<br>R:5'GTGCGTGTCGTGGAGTCG3'	60	66
hsa-miR-4320	GSP:5'GGGGGAAAGGGATTCTGTAG3'<br>R:5'GTGCGTGTCGTGGAGTCG3'	60	64
hsa-miR-4770	GSP:5'GGGGCTTGAGATGACACTG3'<br>R:5'GTGCGTGTCGTGGAGTCG3'	60	62
hsa-miR-340-5p	GSP:5'GCGGTTATAAAGCAATGAGA3'<br>R:5'GTGCGTGTCGTGGAGTCG3'	60	66
hsa-miR-506-3p	GSP:5'GGGATAAGGCACCCTTCTG3'<br>R:5'GTGCGTGTCGTGGAGTCG3'	60	63
hsa-miR-8068	GSP:5'GGGGGTGTTTGTTGTAAGGAT3'<br>R:5'GTGCGTGTCGTGGAGTCG3'	60	65
hsa-let-7b-5p	GSP:5'AGGGGGTGAGGTAGTAGGTTGT3'<br>R:5'GTGCGTGTCGTGGAGTCG3'	60	66
hsa-miR-6851-3p	GSP:5'GGATGGCCCTTTGTACCC3'<br>R:5'GTGCGTGTCGTGGAGTCG3'	60	62
hsa-miR-425-5p	GSP:5'GGGGAATGACACGATCACTC3'<br>R:5'GTGCGTGTCGTGGAGTCG3'	60	65

GSP is the specific primer corresponding to miRNA; R is the primer that matched with RT primer.
