# Supplementary material for: Bone-Metabolism-Related Serum microRNAs to Diagnose Osteoporosis in Middle-Aged and Elderly Women
Source: Diagnostics (Basel). 2022 Nov 19;12(11):2872. doi: 10.3390/diagnostics12112872 (PMC9689310; doi:10.3390/diagnostics12112872)
Supplement: Supplementary file 1 [file diagnostics-12-02872-s001.zip › Supplementary Table S10.pdf]

**Supplementary Table S10. The top 20 target genes and their degree of enrichment (DEmiRNAs)**

| **Gene** | **Degree** | **Gene** | **Degree** |
|---|---|---|---|
| *UBB* | 198 | *PIK3R1* | 67 |
| *TP53* | 110 | *CUL3* | 67 |
| *MAPK1* | 79 | *H2AFZ* | 66 |
| *POLR2F* | 79 | *CUL2* | 66 |
| *BTRC* | 79 | *PPP2CA* | 65 |
| *SKP2* | 77 | *UBE2D2* | 64 |
| *RHOA* | 76 | *FBXL7* | 64 |
| *CDC27* | 70 | *CCNB1* | 63 |
| *SMURF1* | 68 | *FBXW7* | 62 |
| *ANAPC7* | 68 | *FBXL3* | 62 |

DEmiRNAs, differentially expressed miRNAs.
