# Supplementary material for: Bone-Metabolism-Related Serum microRNAs to Diagnose Osteoporosis in Middle-Aged and Elderly Women
Source: Diagnostics (Basel). 2022 Nov 19;12(11):2872. doi: 10.3390/diagnostics12112872 (PMC9689310; doi:10.3390/diagnostics12112872)
Supplement: Supplementary file 1 [file diagnostics-12-02872-s001.zip › Supplementary Table S2.pdf]

Supplementary Table S2. Upregulated and downregulated miRNAs between PMOP patients and n-PMOP controls

Level	Gene				
Upregulated miRNAs (*n* = 148)	hsa-miR-5186 (6.57)	hsa-miR-4527 (6.42)	hsa-miR-144-5p (6.41)	hsa-miR-4320 (5.90)	hsa-miR-4770 (5.80)
	hsa-miR-340-5p (5.80)	hsa-miR-506-3p (5.71)	hsa-miR-8068 (5.52)	hsa-let-7b-5p (5.42)	hsa-miR-6851-3p (5.39)
	hsa-miR-136-5p (5.38)	hsa-miR-6737-3p (5.17)	hsa-miR-4504 (5.15)	hsa-miR-6889-3p (5.07)	hsa-miR-4777-5p (5.04)
	hsa-miR-605-3p (5.04)	hsa-miR-6514-3p (4.84)	hsa-miR-128-2-5p (4.74)	hsa-miR-128-1-5p (4.71)	hsa-miR-1298-3p (4.65)
	hsa-miR-103a-3p (4.62)	hsa-miR-5191 (4.61)	hsa-miR-3654 (4.60)	hsa-miR-545-5p (4.57)	hsa-miR-3653-5p (4.56)
	hsa-miR-15b-5p (4.56)	hsa-miR-4658 (4.53)	hsa-miR-491-3p (4.52)	hsa-miR-4490 (4.51)	hsa-let-7c-5p (4.48)
	hsa-miR-4690-5p (4.47)	hsa-let-7a-5p (4.36)	hsa-miR-5010-3p (4.32)	hsa-miR-107 (4.22)	hsa-miR-6880-3p (4.19)
	hsa-miR-4692 (4.18)	hsa-miR-144-3p (4.17)	hsa-miR-8086 (4.08)	hsa-miR-299-3p (4.05)	hsa-miR-21-3p (4.03)
	hsa-miR-1245b-3p (4.02)	hsa-miR-216b-5p (3.78)	hsa-miR-6506-3p (3.76)	hsa-miR-507 (3.74)	hsa-miR-99b-3p (3.64)
	hsa-miR-4694-3p (3.50)	hsa-miR-1208 (3.49)	hsa-miR-1303 (3.49)	hsa-miR-183-5p (3.49)	hsa-miR-200b-3p (3.49)
	hsa-miR-217 (3.49)	hsa-miR-3157-5p (3.49)	hsa-miR-3668 (3.49)	hsa-miR-3913-5p (3.49)	hsa-miR-3914 (3.49)
	hsa-miR-4300 (3.49)	hsa-miR-4538 (3.49)	hsa-miR-4677-3p (3.49)	hsa-miR-4725-5p (3.49)	hsa-miR-490-5p (3.49)
	hsa-miR-5588-5p (3.49)	hsa-miR-580-5p (3.49)	hsa-miR-6760-3p (3.49)	hsa-miR-6832-5p (3.49)	hsa-miR-6870-3p (3.49)
	hsa-miR-922 (3.49)	hsa-miR-937-3p (3.49)	hsa-miR-4793-3p (3.47)	hsa-miR-181b-5p (3.42)	hsa-miR-4763-3p (3.29)
	hsa-miR-520a-3p (3.24)	hsa-miR-7108-5p (3.20)	hsa-miR-6753-5p (3.19)	hsa-miR-3917 (3.15)	hsa-miR-6718-5p (3.04)
	hsa-miR-1266-3p (3.01)	hsa-miR-7157-3p (2.99)	hsa-miR-378j (2.98)	hsa-miR-4434 (2.98)	hsa-miR-4754 (2.87)
	hsa-miR-511-3p (2.86)	hsa-miR-6840-3p (2.83)	hsa-miR-181a-2-3p (2.80)	hsa-miR-500a-3p (2.79)	hsa-miR-6892-3p (2.79)
	hsa-miR-6503-3p (2.77)	hsa-miR-6731-3p (2.77)	hsa-miR-7154-3p (2.72)	hsa-miR-3155a (2.72)	hsa-miR-6800-5p (2.70)

	hsa-miR-4652-5p (2.69)	hsa-miR-6777-5p (2.56)	hsa-miR-2355-5p (2.51)	hsa-miR-4426 (2.49)	hsa-miR-6854-5p (2.36)
	hsa-miR-8075 (2.35)	hsa-miR-4518 (2.33)	hsa-miR-1304-3p (2.25)	hsa-miR-6795-3p (2.25)	hsa-miR-6865-3p (2.23)
	hsa-miR-5003-3p (2.22)	hsa-miR-4431 (2.20)	hsa-miR-3915 (2.16)	hsa-miR-6884-5p (2.11)	hsa-miR-6800-3p (2.06)
	hsa-miR-373-3p (2.02)	hsa-miR-6509-5p (2.00)	hsa-miR-4436b-3p (1.85)	hsa-miR-3648 (1.80)	hsa-miR-541-3p (1.78)
	hsa-miR-1258 (1.77)	hsa-miR-3133 (1.74)	hsa-miR-4784 (1.71)	hsa-miR-4712-3p (1.70)	hsa-miR-30c-2-3p (1.65)
	hsa-miR-6134 (1.63)	hsa-miR-184 (1.54)	hsa-miR-6739-5p (1.51)	hsa-miR-3150b-3p (1.51)	hsa-miR-182-5p (1.49)
	hsa-miR-4673 (1.46)	hsa-miR-1288-5p (1.44)	hsa-miR-492 (1.36)	hsa-miR-4691-3p (1.33)	hsa-miR-6797-5p (1.33)
	hsa-miR-4632-5p (1.31)	hsa-miR-6766-5p (1.30)	hsa-miR-3150b-5p (1.23)	hsa-miR-5587-3p (1.23)	hsa-miR-6888-5p (1.23)
	hsa-miR-4651 (1.21)	hsa-miR-4309 (1.19)	hsa-miR-3132 (1.17)	hsa-miR-6125 (1.16)	hsa-miR-487a-3p (1.16)
	hsa-miR-5197-3p (1.16)	hsa-miR-664a-5p (1.13)	hsa-miR-6717-5p (1.11)	hsa-miR-6791-5p (1.09)	hsa-miR-4306 (1.08)
	hsa-miR-4469 (1.07)	hsa-miR-502-3p (1.07)	hsa-miR-6788-5p (1.06)	hsa-miR-4737 (1.04)	hsa-miR-330-3p (1.04)
	hsa-miR-6745 (1.03)	hsa-miR-1247-3p (1.01)	hsa-miR-6748-5p (1.01)		
Downregulated miRNAs (*n* = 50)	hsa-miR-4767 (–5.11)	hsa-miR-4724-5p (–3.70)	hsa-miR-1260a (–2.90)	hsa-miR-1270 (–2.50)	hsa-miR-6500-3p (–2.47)
	hsa-miR-4260 (–2.38)	hsa-miR-6752-5p (–2.38)	hsa-miR-4444 (–2.27)	hsa-miR-668-5p (–2.27)	hsa-miR-4768-5p (–2.20)
	hsa-miR-6781-5p (–2.18)	hsa-miR-6877-5p (–2.14)	hsa-miR-3158-5p (–1.97)	hsa-miR-921 (–1.97)	hsa-miR-4429 (–1.96)
	hsa-miR-758-5p (–1.84)	hsa-miR-6769a-5p (–1.75)	hsa-miR-4698 (–1.69)	hsa-miR-6798-5p (–1.69)	hsa-miR-6786-5p (–1.69)
	hsa-miR-4261 (–1.64)	hsa-miR-125a-3p (–1.62)	hsa-miR-6798-3p (–1.60)	hsa-miR-4299 (–1.55)	hsa-miR-6738-5p (–1.55)
	hsa-miR-550a-5p (–1.54)	hsa-miR-300 (–1.54)	hsa-miR-328-5p (–1.54)	hsa-miR-616-3p (–1.54)	hsa-miR-92a-1-5p (–1.54)
	hsa-miR-518f-5p (–1.51)	hsa-miR-4687-3p (–1.47)	hsa-miR-4484 (–1.44)	hsa-miR-934 (–1.43)	hsa-miR-4428 (–1.41)

	hsa-miR-494-3p (–1.38)	hsa-miR-6812-5p (–1.37)	hsa-miR-6814-5p (–1.35)	hsa-miR-4667-5p (–1.31)	hsa-miR-574-3p (–1.30)
	hsa-miR-6845-5p (–1.29)	hsa-miR-933 (–1.28)	hsa-miR-4656 (–1.24)	hsa-miR-6893-5p (–1.21)	hsa-miR-7847-3p (–1.19)
	hsa-miR-4788 (–1.15)	hsa-miR-6086 (–1.10)	hsa-miR-6826-5p (–1.09)	hsa-miR-7152-3p (–1.08)	hsa-miR-6794-5p (–1.02)

The screening criteria of DEmiRNAs were adj.$P < 0.05$ and $|Log2FC| > 1$. The values of Log2FC were shown in parentheses.

PMOP, postmenopausal osteoporosis; n-PMOP, postmenopausal without osteoporosis; FC, fold change.
