# Supplementary material for: Bone-Metabolism-Related Serum microRNAs to Diagnose Osteoporosis in Middle-Aged and Elderly Women
Source: Diagnostics (Basel). 2022 Nov 19;12(11):2872. doi: 10.3390/diagnostics12112872 (PMC9689310; doi:10.3390/diagnostics12112872)
Supplement: Supplementary file 1 [file diagnostics-12-02872-s001.zip › Supplementary Table S3.pdf]

Supplementary Table S3. Verification of microarray results by qRT–PCR

Variable	$2^{-\Delta\Delta CT}$	FC	Log2FC	*P*
hsa-miR-5186				
PMOP	2.91	2.51	1.33	**< 0.001**
n-PMOP	1.16			
hsa-miR-4527				
PMOP	5.76	2.77	1.47	**0.036**
n-PMOP	2.08			
hsa-miR-144-5p				
PMOP	66.52	23.97	4.58	**0.005**
n-PMOP	2.78			
hsa-miR-4320				
PMOP	2.05	3.77	1.91	**< 0.001**
n-PMOP	0.55			
hsa-miR-4770				
PMOP	2.31	3.26	1.70	**0.006**
n-PMOP	0.71			
hsa-miR-340-5p				
PMOP	6.02	6.08	2.60	**< 0.001**
n-PMOP	0.99			
hsa-miR-506-3p				
PMOP	8.75	6.47	2.69	**< 0.001**
n-PMOP	1.35			
hsa-miR-8068				
PMOP	7.76	6.19	2.63	**< 0.001**
n-PMOP	1.25			
hsa-let-7b-5p				
PMOP	3.53	3.53	1.82	**0.007**
n-PMOP	1.00			
hsa-miR-6851-3p				
PMOP	5.06	4.95	2.31	**< 0.001**
n-PMOP	1.02			

All *P* values were calculated with the *t*-test. *P* value < 0.05 was considered to indicate a statistically significant difference (highlighted in bold).

qRT-PCR, quantitative real-time PCR; FC, fold change; PMOP, postmenopausal osteoporosis; n-PMOP, postmenopausal without osteoporosis.
