# Supplementary material for: Bone-Metabolism-Related Serum microRNAs to Diagnose Osteoporosis in Middle-Aged and Elderly Women
Source: Diagnostics (Basel). 2022 Nov 19;12(11):2872. doi: 10.3390/diagnostics12112872 (PMC9689310; doi:10.3390/diagnostics12112872)
Supplement: Supplementary file 1 [file diagnostics-12-02872-s001.zip › Supplementary Table S4.pdf]

Supplementary Table S4. qRT–PCR results for candidate key miRNAs in the training set

**Variable**	**$2^{-\Delta\Delta CT}$**	**FC**	**Log2FC**	***P***
hsa-miR-144-5p				
PMOP	1.66	3.02	1.59	**0.028**
n-PMOP	0.55			
hsa-miR-340-5p				
PMOP	0.57	1.44	0.53	0.371
n-PMOP	0.40			
hsa-miR-506-3p				
PMOP	1.14	3.19	1.67	**0.030**
n-PMOP	0.36			
hsa-miR-8068				
PMOP	1.02	1.56	0.64	**0.009**
n-PMOP	0.65			
hsa-miR-6851-3p				
PMOP	1.30	3.09	1.63	**0.009**
n-PMOP	0.42			

All *P* values were calculated with the *t*-test. *P* value < 0.05 was considered to indicate a statistically significant difference (highlighted in bold).

qRT-PCR, quantitative real-time PCR; FC, fold change; PMOP, postmenopausal osteoporosis; n-PMOP, postmenopausal without osteoporosis.
