# Supplementary material for: Bone-Metabolism-Related Serum microRNAs to Diagnose Osteoporosis in Middle-Aged and Elderly Women
Source: Diagnostics (Basel). 2022 Nov 19;12(11):2872. doi: 10.3390/diagnostics12112872 (PMC9689310; doi:10.3390/diagnostics12112872)
Supplement: Supplementary file 1 [file diagnostics-12-02872-s001.zip › Supplementary Table S5.pdf]

Supplementary Table S5. Performance characteristics of the miRNAs for diagnosing PMOP (training set)

Variable	AUC (95% CI)	*P*	Sensitivity (95% CI)	Specificity (95% CI)	PPV (95% CI)	NPV (95% CI)
Numbers of miRNA: 1						
hsa-miR-144-5p	0.747 (0.580–0.874)	0.006	100.0 (85.8–100.0)	50.0 (23.0–77.0)	77.4 (58.9–90.4)	100.0 (59.0–100.0)
hsa-miR-506-3p	0.832 (0.675–0.933)	**< 0.001**	91.7 (73.0–99.9)	78.6 (49.2–95.3)	88.0 (68.8–97.5)	84.6 (54.6–98.1)
hsa-miR-8068	0.775 (0.611–0.894)	**< 0.001**	66.7 (44.7–84.4)	85.7 (57.2–98.2)	88.9 (65.3–98.6)	60.0 (36.1–80.9)
hsa-miR-6851-3p	0.814 (0.655–0.921)	**< 0.001**	91.7 (73.0–99.0)	64.3 (35.1–87.2)	81.5 (61.9–93.7)	81.8 (48.2–97.7)
Numbers of miRNA: 2						
hsa-miR-144-5p + hsa-miR-506-3p	0.804 (0.643–0.914)	**< 0.001**	100.0 (85.8–100.0)	57.14 (28.9–82.3)	80.0 (61.4–92.3)	100.0 (63.1–100.0)
hsa-miR-144-5p + hsa-miR-8068	0.810 (0.650–0.918)	**< 0.001**	70.8 (48.9–87.4)	85.7 (57.2–98.2)	89.5 (66.9–98.7)	63.2 (38.4–83.7)
hsa-miR-144-5p + hsa-miR-6851-3p	0.824 (0.667–0.928)	**< 0.001**	95.8 (78.9–99.9)	57.14 (28.9–82.3)	79.3 (60.3–92.0)	88.9 (51.8–99.7)
hsa-miR-506-3p + hsa-miR-8068	0.801 (0.639–0.912)	**< 0.001**	70.8 (48.9–87.4)	85.7 (57.2–98.2)	89.5 (66.9–98.7)	63.2 (38.4–83.7)
hsa-miR-506-3p + hsa-miR-6851-3p	0.818 (0.660–0.924)	**< 0.001**	91.7 (73.0–99.0)	64.3 (35.1–87.2)	81.5 (61.9–93.7)	81.8 (48.2–97.7)
hsa-miR-8068 + hsa-miR-6851-3p	0.810 (0.650–0.918)	**< 0.001**	79.2 (57.8–92.9)	78.6 (49.2–95.3)	86.4 (65.1–97.1)	68.7 (41.3–89.0)
Numbers of miRNA: 3						
hsa-miR-144-5p + hsa-miR-506-3p + hsa-miR-8068	0.813 (0.653–0.920)	**< 0.001**	75.0 (53.3–90.2)	85.7 (57.2–98.2)	90.0 (68.3–98.8)	66.7 (41.0–86.7)
hsa-miR-144-5p + hsa-miR-506-3p + hsa-miR-6851-3p	0.836 (0.681–0.936)	**< 0.001**	95.8 (78.9–99.9)	57.1 (28.9–82.3)	79.3 (60.3–92.0)	88.9 (51.8–99.7)
hsa-miR-506-3p + hsa-miR-8068 + hsa-miR-6851-3p	0.815 (0.656–0.922)	**< 0.001**	83.3 (62.6–95.3)	71.4 (41.9–91.6)	83.3 (62.6–95.3)	71.4 (41.9–91.6)
hsa-miR-144-5p + hsa-miR-8068 + hsa-miR-6851-3p	0.836 (0.681–0.936)	**< 0.001**	79.2 (57.8–92.9)	78.6 (49.2–95.3)	86.4 (65.1–97.1)	68.7 (41.3–89.0)
Numbers of miRNA: 4						
hsa-miR-144-5p + hsa-miR-506-3p +	0.845 (0.691–0.942)	**< 0.001**	91.7 (73.0–99.0)	64.3 (35.1–87.2)	81.5 (61.9–93.7)	81.8 (48.2–97.7)

**Variable**	**AUC (95% CI)**	***P***	**Sensitivity (95% CI)**	**Specificity (95% CI)**	**PPV (95% CI)**	**NPV (95% CI)**
hsa-miR-8068 + hsa-miR-6851-3p						

Data are presented as value, value (95% CI) or % (95% CI). *P* value < 0.05 was considered to indicate a statistically significant difference (highlighted in bold).

PMOP, postmenopausal osteoporosis; AUC, area under the curve; CI, confidence interval; PPV, positive predictive value; NPV, negative predictive value.
