# Supplementary material for: Bone-Metabolism-Related Serum microRNAs to Diagnose Osteoporosis in Middle-Aged and Elderly Women
Source: Diagnostics (Basel). 2022 Nov 19;12(11):2872. doi: 10.3390/diagnostics12112872 (PMC9689310; doi:10.3390/diagnostics12112872)
Supplement: Supplementary file 1 [file diagnostics-12-02872-s001.zip › Supplementary Table S6.pdf]

Supplementary Table S6. General clinical data, serum BTMs and biochemical indices of participants with different BMDs

Variable	Normal (n = 6)	Osteopenia (n = 20)	Osteoporosis (n = 29)	Severe osteoporosis (n = 18)	F	P
Age (years)	59.2 ± 6.3[d]	61.4 ± 7.8[d]	65.9 ± 9.3	68.7 ± 5.5[ab]	3.930	**0.012**
BMI (g/cm^2)	24.4 ± 2.7	23.7 ± 2.6	22.0 ± 3.2	22.7 ± 3.7	1.734	0.168
Age at menopause (years)	53.5 ± 2.9	51.5 ± 3.7	51.0 ± 3.3	52.6 ± 4.3	1.247	0.299
Menopausal duration (years)	5.7 ± 4.5[cd]	10.0 ± 7.0[cd]	14.9 ± 9.0[ab]	16.2 ± 6.5[ab]	4.555	**0.006**
LS 1–4 BMD (T-score)	0.0 ± 0.9[bcd]	–1.3 ± 0.7[acd]	–2.6 ± 0.9[abd]	–3.5 ± 1.3[abc]	28.679	**< 0.001**
TH BMD (T-score)	–0.1 ± 0.6[bcd]	–1.4 ± 0.6[acd]	–2.3 ± 0.9[ab]	–2.7 ± 0.6[ab]	22.150	**< 0.001**
FN BMD (T-score)	–0.6 ± 0.6[bcd]	–1.8 ± 0.5[acd]	–2.7 ± 0.7[ab]	–2.9 ± 0.6[ab]	31.475	**< 0.001**
LS 1–4 BMD (g/cm^2)	1.110 ± 0.111[bcd]	0.941 ± 0.078[acd]	0.800 ± 0.099[abd]	0.692 ± 0.149[abc]	30.002	**< 0.001**
TH BMD (g/cm^2)	0.962 ± 0.071[bcd]	0.788 ± 0.082[acd]	0.684 ± 0.111[abd]	0.627 ± 0.084[abc]	23.506	**< 0.001**
FN BMD (g/cm^2)	0.864 ± 0.066[bcd]	0.713 ± 0.055[acd]	0.616 ± 0.077[ab]	0.578 ± 0.076[ab]	32.011	**< 0.001**
25(OH)D (ng/mL)	26.7 ± 9.8	23.6 ± 6.4	23.3 ± 6.6	24.3 ± 10.6	0.317	0.813
N-MID (ng/mL)	20.46 ± 6.59	16.79 ± 6.25[c]	21.43 ± 8.69[bd]	15.37 ± 8.15[c]	2.772	**0.048**
P1NP (ng/mL)	57.14 ± 44.01	57.79 ± 29.50	55.48 ± 27.90	49.05 ± 30.41	0.296	0.828
β-CTX (ng/mL)	0.512 ± 0.234	0.526 ± 0.286	0.605 ± 0.518	0.637 ± 0.475	0.283	0.838
UA (μmol/L)	304.0 ± 79.2	300.2 ± 93.4	305.7 ± 73.4	296.3 ± 90.2	0.050	0.985
ALP (U/L)	68.5 ± 17.6[d]	75.6 ± 31.3[d]	74.4 ± 21.6[d]	108.2 ± 36.3[abc]	6.588	**0.001**
Calcium (mmol/L)	2.30 ± 0.19	2.21 ± 0.18[c]	2.32 ± 0.12[bd]	2.21 ± 0.19[c]	2.617	0.058
Phosphorus (mmol/L)	1.34 ± 0.07[bcd]	1.10 ± 0.18[a]	1.17 ± 0.18[a]	1.16 ± 0.19[a]	2.791	**0.047**

Data are presented as mean ± standard deviation. All P values were calculated with the One-Way ANOVA. a, $P < 0.05$ when compared to normal group; b, $P < 0.05$ when compared to osteopenia group; c, $P < 0.05$ when compared to osteoporosis group; d, $P < 0.05$ when compared to severe osteoporosis group.

BTMs, bone turnover markers; BMD, bone mineral density; BMI, body mass index; LS, lumbar spine; TH, total hip; FN, femoral neck; 25(OH)D, 25-hydroxy vitamin D; N-MID, N-terminal middle segment osteocalcin; P1NP, propeptide of type I procollagen; β-CTX, β-C-terminal telopeptide of type I collagen; UA, uric acid; ALP, alkaline phosphatase.
