# Supplementary material for: Bone-Metabolism-Related Serum microRNAs to Diagnose Osteoporosis in Middle-Aged and Elderly Women
Source: Diagnostics (Basel). 2022 Nov 19;12(11):2872. doi: 10.3390/diagnostics12112872 (PMC9689310; doi:10.3390/diagnostics12112872)
Supplement: Supplementary file 1 [file diagnostics-12-02872-s001.zip › Supplementary Table S7.pdf]

**Supplementary Table S7. The top 10 upregulated and downregulated DEmiRNAs and their target genes**

| Level | Gene | Target gene |
|---|---|---|
| Upregulated | hsa-miR-5186 | - |
| | hsa-miR-4527 | - |
| | hsa-miR-144-5p | *SYNPO2* |
| | hsa-miR-4320 | *PABPN1, BCL2L2-PABPN1* |
| | hsa-miR-4770 | - |
| | hsa-miR-340-5p | *ACER3, SNX5, SKP2, DEGS1, FBXL3, ZXDB, LAPTM4A, PPIP5K2, LIMS1, KCNK1, ZXDA, RPS6KA5, CC2D2A, SLITRK4, TMEM245, CAPRIN2, FIGN, NEK7, ELAVL4, SCARB2, KIAA0895, REV3L, GK5, KDELC2, ERH, BTBD10, LCLAT1, SHCBP1, DLC1, RASEF, OCLN, IPO7, DDX5, MED17, UBXN2A, BCOR, MYLIP, CALM1, KIAA1161, KLF10, UBE2Z, INO80D, APPL1, CREBRF, SLC7A11, ANKRD29, KAT6B, LEPROT, YY1, PDE4D, SIVA1, MAT2A, PUM1, SLC24A2* |
| | hsa-miR-506-3p | *SCAMP4, PARP16, NEK9, TMEM41A, ZWINT, MYO10, AMOTL1, PTBP3, CREBRF, SNX18, PI4K2B, CHSY1, LRRC58, LRRC1, VIM, SNAI2, CD151, SLC16A1, PTBP1, PRR14L, NUFIP2, GXYLT1, SFT2D3* |
| | hsa-miR-8068 | *ENOX2, ZBTB18, KIAA0408, YWHAE, USP14, 1-Mar, PDPK1, PURB, G2E3, UBN2, TTC26, SLC3A2, ZNF207, SEMA6D, DMD* |
| | hsa-let-7b-5p | *NR6A1, CHD4, PDP2, PALD1, PDPR, BZW1, CBX5, SLC25A24, CEP135, LIN28B, SLC5A6, IGF1R, MAPK6, NME6, NAP1L1, RAB11FIP4, KLHDC8B, ACER2, MBD2, CCNF, TGFBR1, PLAGL2, C19orf47, RIOK3, LIMD2, BACH1, USP38, TRIM71, GNG5, HAND1, CDC25A, ZBTB5, ARPP19, NME4, PPP1R15B, CDKN1A, MTUS1, FAM104A, PRKAA2, PEX11B, SMC1A, CLDN12, NHLRC3, COL3A1, TMEM2, RDH10, BZW2, RALB, CDC34, CPA4, IGF2BP3, ANKRD46, SPRYD4, ESPL1, AP1S1, DMD, GRPEL2, NAA30, NRAS, RAB3GAP2, MDM4, KMT2D, PCGF3, LRIG3, EDEM3, TMED5, SOCS1, BTBD9, IRS2, MIB1, TAF9B, E2F5, PGRMC1, FNDC3A, SOX13, SLC25A32, NXT2, AHCTF1, KCTD21, TGFBR3, IGF2BP2, MXD1, SMARCAD1, DPF2, HMGA2, TMEM167A, E2F6, SMARCC1, FAM84B, YOD1, ZCCHC3, SMCR8, COIL, KIAA0930, CPEB1, CCNJ, RRM2, SEMA4C, CRY2, SLC20A1, HMGA1, PLXND1, EDN1, MARS2, ZNF644, ZNF566, RDX, SLC10A7, LBR, IGDCC4, IGF2BP1, SCD, FIGN, UTRN, DNA2, PRSS22, EIF4G2* |
| | hsa-miR-6851-3p | *NAA15, GTPBP2* |

| Level | Gene | Target gene |
|---|---|---|
| Downregulated | hsa-miR-4767 | *SRP54* |
| | hsa-miR-4724-5p | *FOXN2, CTBP2, HSP90AA1, STK4, ATP1B3, IRF2BP2, SLC35G2, SPATA2,, RAD51AP1, YTHDC1, PCBP1* |
| | hsa-miR-1260a | *TAF8, ATF6B, ORMDL2, TFDP2, KIF21A, SCD, TSC22D2, KIF3A, ICMT, MKNK2*<br>*GATAD2B* |
| | hsa-miR-1270 | *YWHAQ, OLA1, IRF2BP2* |
| | hsa-miR-6500-3p | *CCDC71L, DCC, BRAP, ZNF117, ZNF43, ZC3H8, FOXN3* |
| | hsa-miR-4260 | *IL21R, UBB, ZNF215, FXR2* |
| | hsa-miR-6752-5p | *SETD1B, MID1IP1, KHSRP, BCL3, RUNX3, PPP6R1, CLASP1, KCNK3, FXR2, RREB1, DCX, GRIN2D, PLEC, RAB2A, ZNF436, GIGYF1, OTUB1* |
| | hsa-miR-4444 | - |
| | hsa-miR-668-5p | *CDC25A* |
| | hsa-miR-4768-5p | *NEURL1B, ASCL1, ZNF468, ZNF611, LPP, ZNF594, ARF6, TRUB1, ZNF208, SPOP, USP37, KLHL28, HSF5, ZNF701, USP32, ZNF439, ANKRD28, LSAMP, CHD9, SNRK, ZNF207, ZNF90, LZIC, SLCO3A1, ZNF117, CADM2, SLC6A8, POU2F1, ZNF415, LRP6, GATM, ATP1A3, FBXL20, ZNF99, NFIX* |

-, No target gene was predicted.

DEmiRNAs, differentially expressed miRNAs.
