# Supplementary figures and images for: Bone-Metabolism-Related Serum microRNAs to Diagnose Osteoporosis in Middle-Aged and Elderly Women
Source: Diagnostics (Basel). 2022 Nov 19;12(11):2872. doi: 10.3390/diagnostics12112872 (PMC9689310; doi:10.3390/diagnostics12112872)

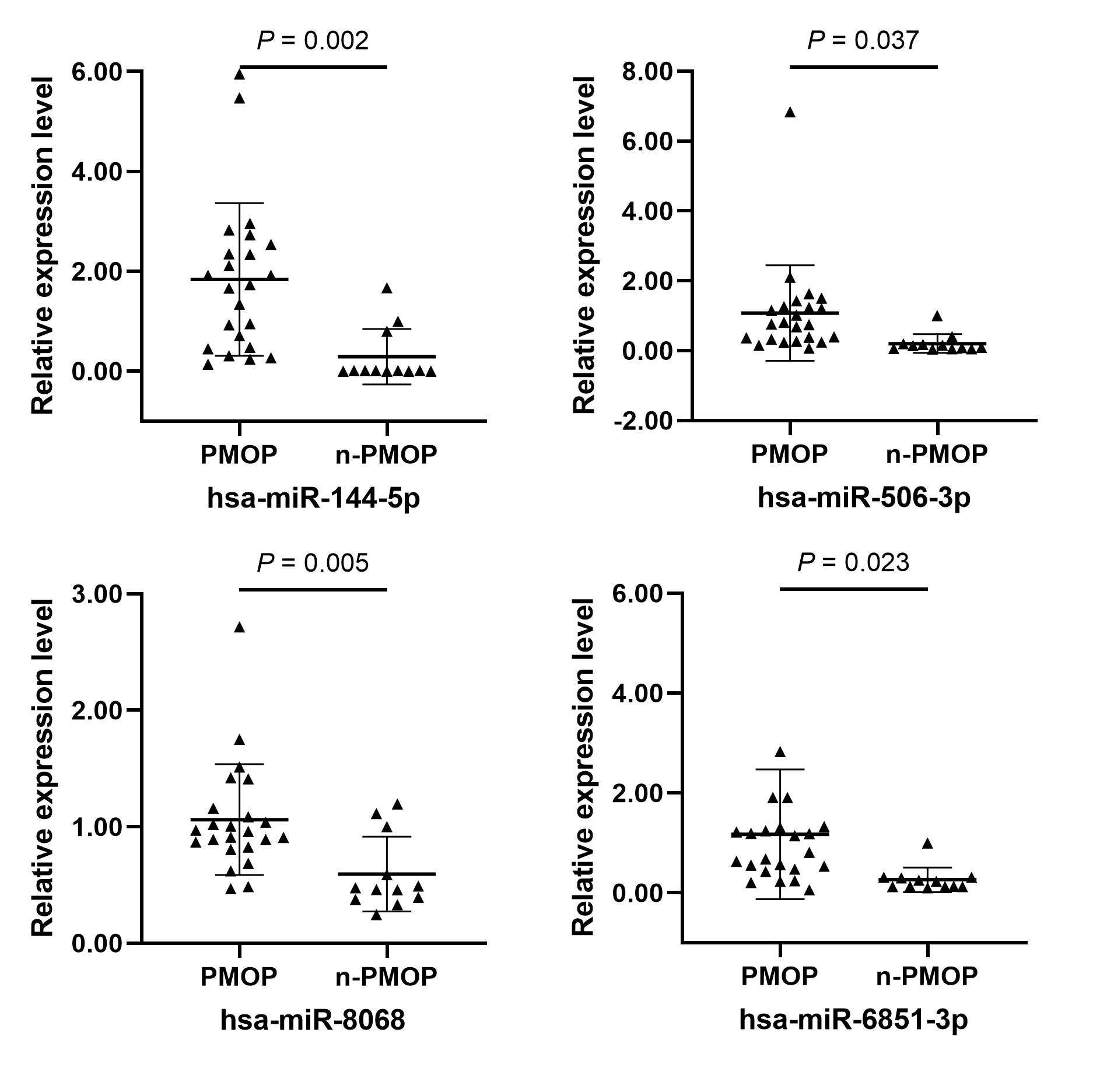

Supplement: Supplementary file 1 [file diagnostics-12-02872-s001.zip › Supplementary Figure S1.tif]

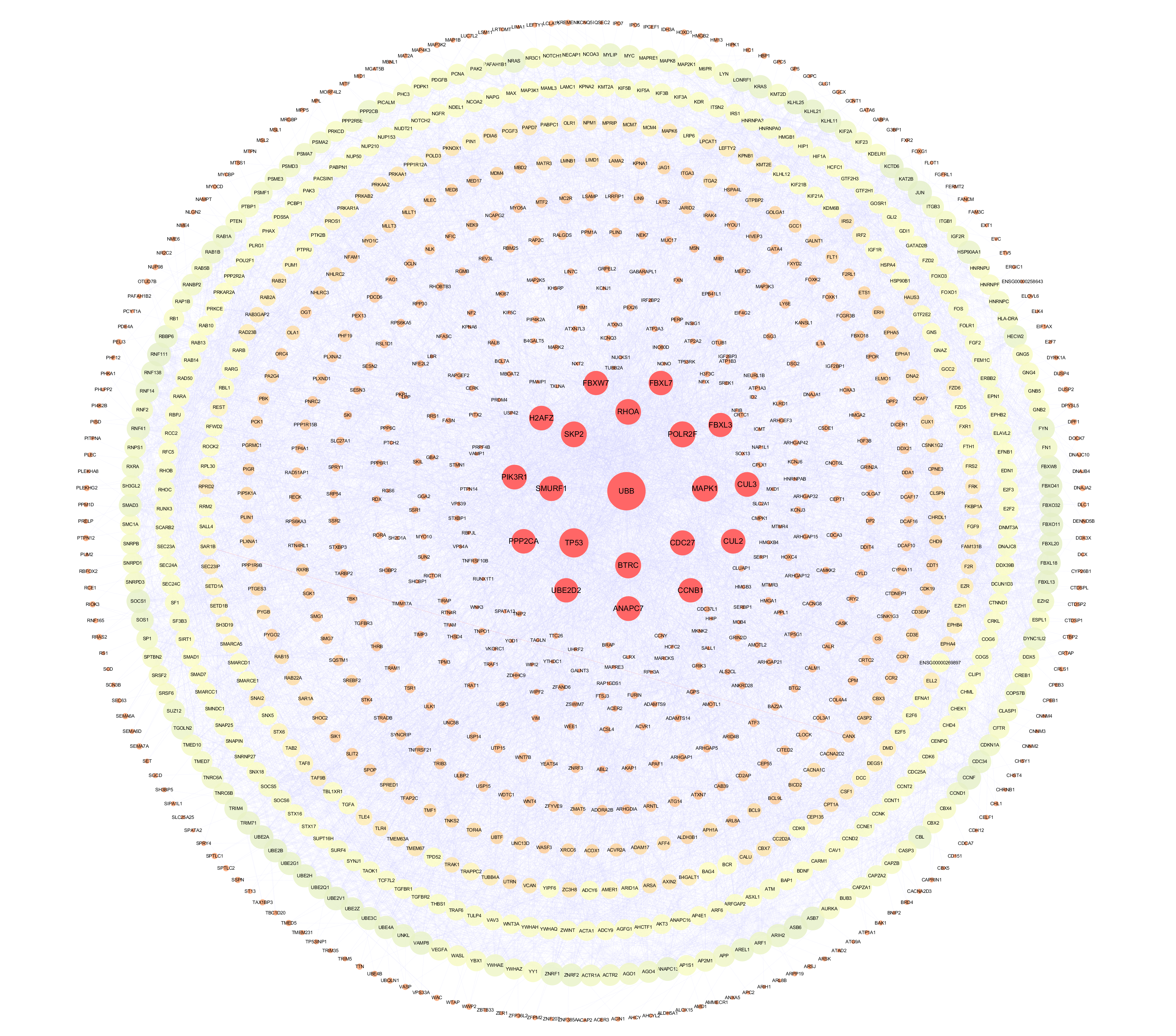

Supplement: Supplementary file 1 [file diagnostics-12-02872-s001.zip › Supplementary Figure S2.png]
